# A Systematic Review and Meta-Analysis of the Role of Open Platysmaplasty in Facelift and Necklift Surgery: Comparative Outcomes of Closed- vs Open-Neck Rejuvenation With Liposuction Using FACE-Q Scales

**DOI:** 10.1093/asjof/ojag140

**Published:** 2026-07-02

**Authors:** Giuseppe Fiore, Naghmeh Naderi, Benedict Reed, Lauren Andrea Laird, Rana Das-Gupta

## Abstract

Facial and neck rejuvenation techniques have evolved significantly, aiming to achieve optimal patient satisfaction with minimal invasiveness. Open platysmaplasty techniques allow direct access to anatomical structures but involve additional scarring, whereas closed techniques are less invasive but might provide limited correction. There remains uncertainty regarding patient-reported outcomes between these approaches. This systematic review and meta-analysis assessed patient-reported outcomes (FACE-Q Aesthetic Module scores) comparing open- vs closed-neck rejuvenation techniques, with and without liposuction. A systematic review was performed following Preferred Reporting Items for Systematic Reviews and Meta-Analyses 2020 guidelines, searching databases (MEDLINE [Ovid], Embase [Ovid], PubMed, and Cochrane Library) from inception to November 2024. Studies comparing open- and closed-necklift techniques and reporting FACE-Q scores were included. Data were analyzed using weighted means, pooled standard deviations, and Welch's *t*-tests to compare satisfaction scores. Nine studies with 847 patients were analyzed. When liposuction was employed, open techniques significantly improved neck aesthetic satisfaction (mean FACE-Q = 81.5) compared with closed techniques (mean FACE-Q = 77.5, *P* = .022), although chin scores were comparable (*P* = .268). Closed techniques without liposuction yielded the highest satisfaction scores for both chin (mean FACE-Q = 86.4) and neck (mean FACE-Q = 86.6), significantly exceeding open techniques with liposuction (*P* < .01). Closed-neck rejuvenation techniques without liposuction showed the highest patient-reported outcomes for appropriately selected patients, emphasizing the importance of individualized surgical planning. Open techniques retain significant value for patients requiring more extensive anatomical correction.

Level of Evidence: 4 (Therapeutic)

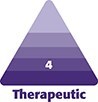

The pursuit of a youthful and well-defined facial and cervical contour has long been a central goal in aesthetic plastic surgery. Multiple age-related changes in the face, including weakened collagen, thinner skin, and the influence of muscular forces—contribute to wrinkle formation. Additionally, lax retaining ligaments and shifting fat compartments cause bulges and accentuate compartmentalization.^1^ These processes are further compounded by muscle atrophy, bone resorption, and loss of skeletal support, culminating in the characteristic manifestations of facial aging.

Facelift surgery has evolved markedly over the past 4 decades. Early approaches largely involved limited skin elevation without significant manipulation of deeper structures such as the superficial musculoaponeurotic system (SMAS). In contrast, contemporary facelift techniques often incorporate dual-plane dissection to address both skin and SMAS, providing enhanced structural support and potentially longer-lasting outcomes. Nevertheless, no single method has emerged as superior for all patient anatomies and aesthetic goals. Facelifts remain among the top 5 most commonly performed aesthetic procedures in the United States where surgeons must balance the invasiveness of the operation and potential complications against recovery times and overall results.^2^

Neck appearance is a critical aspect of facial rejuvenation, given that skin laxity, jowling, and blunting of the cervicomental angle are frequent concerns. Ellenbogen and Karlin's guidelines for an aesthetically appealing neck—which include a well-defined mandibular border, a cervicomental angle of ∼105°– to 120°, and a subhyoid depression—continue to guide surgical decision making.^3,4^

Necklift approaches vary considerably, from open techniques with a submental incision that enables direct platysmal manipulation to closed approaches that avoid a midline incision altogether. Although closed approaches often feature shorter operative times, less scarring, and faster recoveries, there is ongoing debate as to whether they can produce the same comprehensive correction as open techniques—particularly for patients with prominent platysmal banding or significant skin laxity.^5,6^

Historically, facelift outcomes have been assessed through clinical observation, photographic analysis, and complication rates. Increasingly, however, patient-reported outcome measures are recognized for their ability to capture patient satisfaction and overall quality of life. Since 2010, the FACE-Q Aesthetic module has gained prominence as a validated instrument for evaluating facial aesthetic procedures.^7^ Approved by the US FDA as a Medical Device Development Tool, the FACE-Q provides a structured framework for comparing surgical approaches based on patient experiences.^8-10^

In this systematic review and meta-analysis, we compared open- and closed-neck facelift techniques as they apply to cervicofacial rejuvenation. Open-neck facelifts, conducted with a submental incision, allow direct exposure of anatomical structures such as the platysma, digastric muscles, and subplatysmal fat. Standard procedures include midline platysmaplasty, submental liposuction, and selective submandibular gland resection, sometimes with additional measures like quilting sutures to minimize hematoma.^11-13^

In contrast, closed-neck facelifts avoid the submental incision and generally employ lateral platysma suspension anchored to the mastoid fascia, along with various SMAS techniques (plication, dissection, or SMASectomy). In these procedures, submental contouring may be achieved indirectly through lateral liposuction, coupled with soft-tissue redraping through a periauricular incision.

The FACE-Q instrument offers an ideal method to compare these 2 techniques by capturing a comprehensive range of patient perspectives, thereby providing quantitative data on satisfaction and quality of life.

## Study Aim

The primary objective of this systematic review and meta-analysis is to assess whether a closed-neck approach with or without submental liposuction can achieve patient-reported outcomes (FACE-Q scores) comparable to those of an open neck in patients undergoing periauricular facelift and necklift surgery.^7,8^

Specifically, we address 2 key comparisons:

1. When liposuction is employed, does a closed-neck approach suffice as an alternative to open-neck surgery in periauricular facelift/necklift?
2. Without liposuction, is a closed-neck approach an adequate substitute for open-neck surgery in periauricular facelift/necklift?

By emphasizing patient-reported outcomes, this review aims to determine whether less invasive techniques can produce results comparable to more traditional open approaches—ultimately guiding surgeons toward strategies that optimize patient satisfaction, minimize complications, and streamline postoperative recovery.

## METHODS

This systematic review and meta-analysis were conducted in accordance with the Preferred Reporting Items for Systematic Reviews and Meta-Analyses (PRISMA) 2020 guidelines (Supplemental Figure 1).^14^

### Types of Studies

This review included original studies published in English, including cohort studies and observational studies. Eligible studies evaluated patient-reported satisfaction using the FACE-Q Aesthetics module in adult patients (≥18 years) undergoing facelift and/or necklift surgery, incorporating either closed-neck or open-neck techniques.

### PICO framework

To guide study selection and ensure methodological consistency, the research question was structured using the PICO framework.^15^

Population (P): Adult patients (≥18 years) undergoing facelift and/or necklift surgery involving either open-neck or closed-neck techniques, with mild-to-severe neck laxity or platysmal banding.
Intervention (I): Closed-neck facelift techniques (without a submental incision), performed with or without submental liposuction.
Comparison (C): Open-neck facelift techniques involving a submental incision, typically combined with submental liposuction.
Outcome (O): Primary Outcome: Patient-reported satisfaction and aesthetic outcomes assessed using the FACE-Q Aesthetics module at any postoperative time point.

### Search Strategy

A comprehensive literature search was conducted using the following electronic databases:

1. MEDLINE (Ovid)
2. Embase (Ovid)
3. PubMed
4. Cochrane Library

The search period spanned from 1946 to November 2024. Relevant search terms included combinations of “FACE-Q,” “Neck lift,” “Necklift,” “Platysmaplasty,” “Face lift,” “Facelift,” “Neck rejuvenation,” “Necklift and facelift,” “Neck lift and face lift,” “Neck lift and FACE-Q,” “Necklift and FACE-Q,” “Face lift and FACE-Q,” “Facelift and FACE-Q,” “FACE-Q and Neck rejuvenation,” “Cervicoplasty,” “FACE-Q and Cervicoplasty,” “Open neck,” “Closed neck,” and “FACE-Q and open neck.” Boolean operators and MeSH terms were used to optimize the search. The sources were last accessed on November 29, 2024.

### Eligibility Criteria

#### Inclusion Criteria

1. Original peer-reviewed articles, human studies, published in English
2. Studies involving adult patients (≥18 years) undergoing facelift and/or necklift surgery, using either open-neck or closed-neck techniques
3. Studies that used the FACE-Q Aesthetics module as the primary outcome measure, administered at any time point following surgery

#### Exclusion Criteria

1. Nonsurgical studies
2. Studies without neck involvement
3. Studies with incomplete, missing, or irrelevant FACE-Q data
4. Case reports, review articles, conference abstracts, commentaries, or book chapters
5. Non-English language publications
6. Animal studies

### Study Selection and Screening

All titles, abstracts, and full texts were screened independently and in duplicate by 2 reviewers (G.F. and N.N.) using piloted screening forms. Discrepancies or conflicts were resolved through discussion and consensus.^16^

### Outcomes

Neck appearance and patient-centered outcomes were assessed using the FACE-Q Aesthetic Module, specifically focusing on the “Appraisal of Neck” and “Area Under the Chin” scales. The FACE-Q “Area Under the Chin” scale was analyzed alongside “Appraisal of Neck” because the submental region and cervicomental angle form a contiguous aesthetic unit and are directly impacted by fat management. Additionally, several included studies reported “Area Under the Chin” scores but did not report neck-only scores, so analyzing both domains maximized usable evidence. These validated patient-reported outcome measures were used to evaluate aesthetic satisfaction following periauricular facelift and necklift procedures.

The primary outcomes extracted included:

1. Mean postintervention FACE-Q score
2. Standard deviation (SD)

Duration of follow-up (as reported by each study's primary time horizon).^9^

Outcomes were analyzed based on the type of surgical approach, categorized as follows:

1. Open-neck facelift: Performed with a submental incision and combined with liposuction
2. Closed-neck facelift: Performed without a submental incision, either with or without submental liposuction. No included studies reported FACE-Q results for open-neck surgery performed without liposuction or fat removal as a separate group; therefore, this category could not be analyzed separately.

The FACE-Q Aesthetic module scores were derived from ordinal responses and converted to a continuous 0 to 100 scale, in which higher scores represent greater patient satisfaction. The scoring conversion was performed using the standard algorithm:

“Not at all” = 4
“A little” = 3
“Moderately” = 2
“Extremely” = 1

The raw summed scale scores were then transformed using the FACE-Q Rasch transformation table, resulting in final scores ranging from 0 (worst outcome) to 100 (best outcome).^17^

### Data Extraction

Data were extracted and recorded in Microsoft Excel and independently reviewed by 2 reviewers (G.F. and B.R.). Any discrepancies were resolved by consensus, with input from a third reviewer when necessary. For each included study, postintervention FACE-Q data were extracted separately for each intervention group.^18^

The following variables were recorded: first author, year of publication, journal, procedure characteristics, postintervention FACE-Q scores (means and SDs), and the time point at which FACE-Q outcomes were assessed. We extracted the facelift approach and any reported traction vector/fixation strategy at the SMAS–platysma junction when available. We attempted to extract and compare concomitant procedures across cohorts; however, reporting was insufficient to permit stratified or adjusted analyses. Given the high frequency of concurrent liposuction among patients undergoing open-neck facelift procedures, liposuction appears to be routinely incorporated as a standard adjunct to these surgeries. To ensure methodological consistency and minimize potential confounding, studies involving mixed cohorts of patients with and without liposuction were excluded from the final analysis. For open-neck cohorts, we additionally extracted any reported adjunct maneuvers beyond liposuction/fat debulking (eg, platysmaplasty/plication, subplatysmal fat excision, digastric modification, and/or submandibular gland contouring) when available.

### Risk of Bias

Risk of bias was systematically assessed using criteria adapted from Murad et al for observational studies.^19^ Key evaluation domains included patient selection, exposure and outcome ascertainment, completeness of follow-up, and reporting clarity. All included studies were observational, lacking randomization, and introducing inherent selection and performance biases.

### Statistical Methods

Study-level summary statistics (mean FACE-Q scores, SDs, and sample sizes) were extracted directly from the included studies. Data are presented as individual study means and SDs as well as weighted means and SDs of each group of studies. Weighted means account for differences in study size, and pooled SDs permit estimation of within-group variability. Comparisons are made between open and closed approaches when used in conjunction with liposuction as well as comparing a closed-neck approach with an open-neck approach with liposuction. Studies are separated by their anatomical FACE-Q score with studies either reporting this score for the neck and/or chin. Comparative analysis uses Welch's *t*-tests as the groups have unequal sample sizes and/or variances. A *t*-statistic and corresponding degrees of freedom were calculated. A threshold of *P* < .05 is considered statistically significant.^20^

### Rationale for Statistical Methods

Welch's *t*-test was employed for comparative analysis because of its suitability in situations where sample sizes and variances differ between groups—a characteristic frequently observed in the observational studies included. In contrast to the traditional Student's *t*-test, Welch's version does not require the assumption of equal variances, making it more appropriate for analyses involving heterogeneous data.

To account for varying study sizes, weighted means and pooled SDs were used. This method gives greater influence to larger studies, thereby providing a more balanced and representative estimate of central tendency and variability across the dataset. This weighting enhances the overall robustness and reliability of the analysis.

## RESULTS

**Figure 1.**
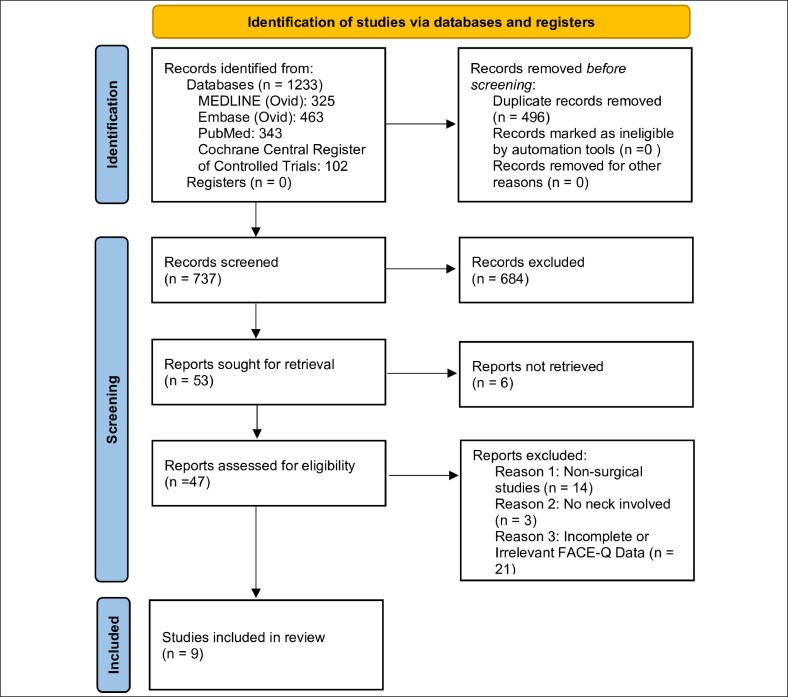
The Preferred Reporting Items for Systematic Reviews and Meta-Analyses flow diagram of study selection.

One thousand two hundred and thirty-three records were identified from Databases (MEDLINE, Embase, PubMed, and Cochrane). After duplicate records were removed, 737 records were screened. The total number of patients who contributed FACE-Q outcome data was 847. Subsequently, 9 studies were included in this review. A detailed overview can be found in PRISMA 2020 flow diagram (Figure 1).

### Chin: Open Neck With Liposuction vs Closed Neck With Liposuction

**Figure 2.**
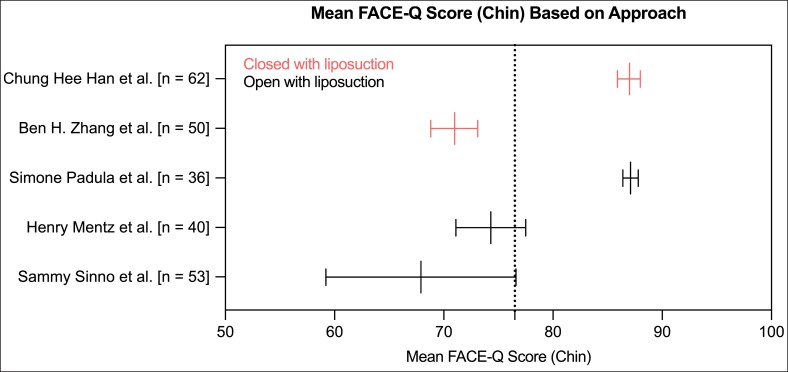
Forest plot comparing Chin-FACE-Q score when using an open or closed approach in conjunction with liposuction. Error bars represent ±standard deviation. The vertical dotted line represents the weighted pooled mean FACE-Q score.

Regarding appraisal of the chin, 5 studies assessed the FACE-Q scores of either open or closed facelift approaches when used in conjunction with liposuction. The closed group (2, *n* = 92) had a weighted mean Chin-FACE-Q score of 78.3 (pooled SD = 6.19) compared with 75.2 (pooled SD = 21.54) in the open group (3, *n* = 129). This difference was not statistically significant (*t* = 1.52, *P* = .129; Figure 2).

### Neck: Open Neck With Liposuction vs Closed Neck With Liposuction

**Figure 3.**
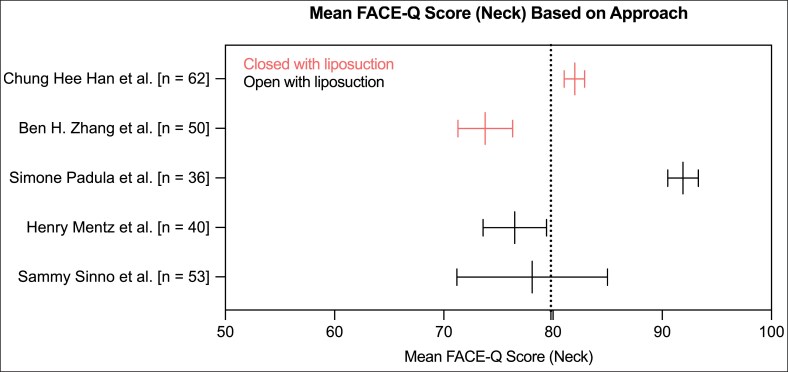
Forest plot comparing Neck-FACE-Q scores when using an open or closed approach in conjunction with liposuction. Error bars represent ±standard deviation. The vertical dotted line represents the weighted pooled mean FACE-Q score.

With regards to appraisal of the neck, 2 closed-neck studies with liposuction (*n* = 92) assessed the FACE-Q and were compared with 3 open-neck studies (*n* = 129). The closed group had a weighted mean FACE-Q score of 77.5 (pooled SD = 6.98) compared with 81.5 (pooled SD = 17.40) in the open group (*n* = 129). This difference was statistically significant (*t* = −2.31, *P* = .022) favoring open-neck approaches for improved neck contour satisfaction when liposuction is available (Figure 3).

### Chin: Open Neck With Liposuction vs Closed Neck Without Liposuction

**Figure 4.**
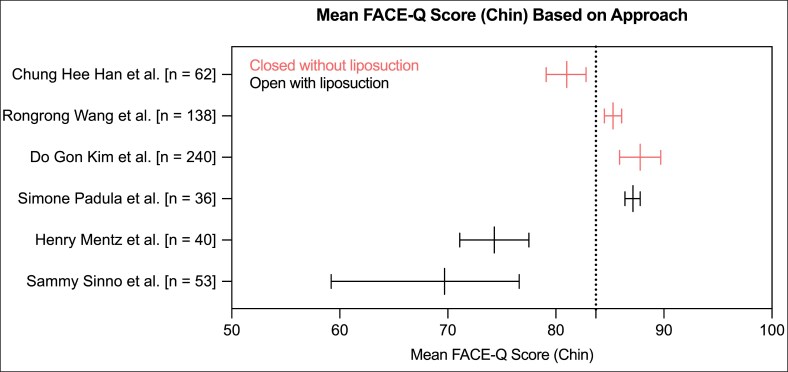
Forest plot comparing Chin-FACE-Q scores between open-with-liposuction and closed-without-liposuction approaches. Error bars represent ±standard deviation. The vertical dotted line represents the weighted pooled mean FACE-Q score.

When considering appraisal of the chin, 3 studies assessed closed approaches without liposuction (*n* = 476) with a pooled mean FACE-Q of 85.3 (pooled SD = 12.8). These are compared with 3 open-with-liposuction studies (*n* = 129, mean = 75.4, pooled SD = 21.5). This difference was statistically significant (*t* = 5.01, *P* = .0001) favoring closed approaches without liposuction for chin contour satisfaction over an open approach with liposuction (Figure 4).

### Neck: Open Neck With Liposuction vs Closed Neck Without Liposuction

For appraisal of the neck, 3 studies contributed data to the closed (*n* = 398, mean = 86.6, pooled SD = 14.7) group and a further 3 studies contributed data to the open-with-liposuction (*n* = 129, mean = 81.5, pooled SD = 17.4) group. The difference was statistically significant (*t* = 3.03, *P* = .003), again favoring the less invasive and less labor-intensive closed approach over an open-with-liposuction facelift.

**Figure 5.**
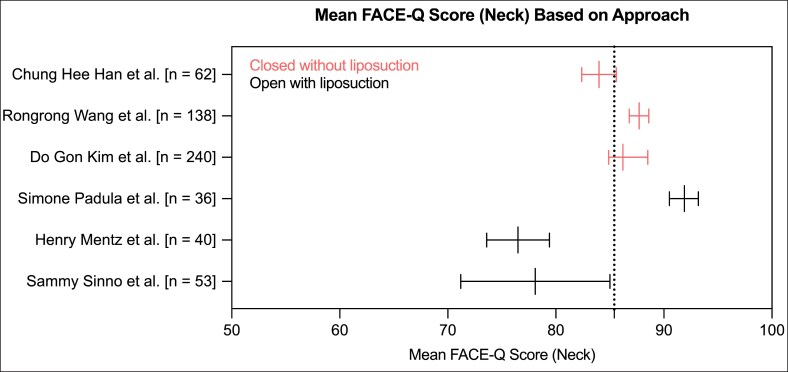
Forest plot comparing Neck-FACE-Q scores between open-with-liposuction and closed-without-liposuction approaches. Error bars represent ±standard deviation. The vertical dotted line represents the weighted pooled mean FACE-Q score.

In summary, when liposuction is used, patients prefer an open-neck approach, although this study shows greatest patient satisfaction with a closed approach without liposuction (Figure 5).

### Characteristics of Included Studies

Kappos et al prospectively evaluated 67 patients (mean age 63 years; range, 54-85 years) who underwent a closed-neck SMAS facelift and necklift without submental liposuction. Using a standard periauricular approach, patients were divided into 3 treatment groups: skin-only lift (*n* = 13), lift with autologous fat lipofilling (*n* = 26), and a “Face-Lift Plus” protocol (*n* = 28) that included upper or lower blepharoplasty and dermabrasion. At a mean follow-up of 61 months (range, 9-108 months), FACE-Q outcomes showed high satisfaction in the lipofilling group (mean “Appraisal of Chin” = 100 ± 6, range, 76-100) and moderately high satisfaction in the Face-Lift Plus group (mean = 83 ± 18, range, 28-100). No FACE-Q scores were reported for the skin-only group. An overall score of 78/100 was provided for the “Area Under Chin” domain. Postoperative complications were minimal and included isolated hematoma, minor infection, and transient facial nerve paresis, with no permanent sequelae. Although the extended follow-up and use of validated patient-reported outcomes strengthen the study, its nonrandomized design, incomplete data for 1 cohort, and lack of blinded photographic assessment limit the generalizability of its conclusions.^21^

Sinno et al reported an observational cohort of 53 consecutive female patients (mean age 63 ± 6.1 years; range, 50-80 years) who underwent a high, extended-SMAS facelift and necklift performed through standard periauricular incisions combined with an open-neck submental approach that incorporated midline platysma plication and adjunct liposuction. At a mean follow-up of 61 months (range, 3-72 months), validated FACE-Q outcomes showed favorable patient-reported aesthetics: the mean “Appraisal of Neck” score reached 78.1 ± 25.6, whereas the mean “Appraisal of the Area Under the Chin” was 67.9 ± 32.3 (0-100 scale; *n* = 53 for both domains). Although the series benefits from long-term follow-up and use of a patient-reported outcome measure, interpretation is tempered by its single-arm design, exclusively female sample, the absence of ethnicity data, and lack of detailed complication reporting or blinded photographic assessment.^22^

Han et al published a 2024 single-center observational series of 62 Asian patients (60 women, 2 men; mean age 48.6 years; range, 40-64 years) who underwent an extended deep-plane rhytidectomy through periauricular incisions with no submental incision or midline platysmaplasty (closed-neck approach). Patients were prospectively stratified into 2 cohorts: Group 1 received the lift alone (*n* = 20), whereas Group 2 underwent the same lift augmented by 1064 nm Nd:YAG laser-assisted liposuction of the lower face (SmartLipo; *n* = 42). At 12 months, validated FACE-Q scores (0-100 scale) showed comparable neck satisfaction between groups (mean “Appraisal of Neck” 84 ± 3.7 vs 82 ± 3.2) but higher submental satisfaction when liposuction was added (mean “Area Under Chin” 87 ± 3.4 vs 81 ± 4.2). No hematomas, thermal injuries, skin necrosis, or nerve paresis occurred; minor sequelae included transient submental firmness (5/62, 8.1%) and slight early asymmetry (6/62, 9.7%), all resolving spontaneously or with a single triamcinolone injection. The study suggests that adding laser-assisted liposuction can enhance chin-neck contour scores without increasing morbidity, although its nonrandomized design and small cohort sizes limit definitive comparative inferences.^23^

Wang et al described a single-center observational series of 138 consecutive female patients (mean age 55.2 ± 9.8 years; range, 39-70 years) from Wuhan, China, who underwent a closed-neck facelift without liposuction. The operation was performed entirely through standard periauricular incisions and centered on a periauricular purse-string suture that plicated the SMAS, elevated the malar fat pad and cranially anchored the platysma to the mastoid-process periosteum, thereby obviating any submental incision. At a mean follow-up of 12 months (range, 6-24 months), patient-reported FACE-Q scores showed very high satisfaction: Appraisal of Neck averaged 87.7 ± 5.3 and Appraisal of the Area Under the Chin averaged 85.3 ± 4.6 (0-100 scale). Overall morbidity was low (6.4%), limited to 3 hypertensive hematomas (2.4%), 3 small areas of skin slough (2.4%) and 2 widened scars (1.6%); no facial-nerve injury or need for surgical reexploration was recorded. Although these data support the efficacy and safety of a purse-string, SMAS-reinforced lift executed through a closed approach, interpretation is tempered by the all-female cohort, absence of a comparison group, and lack of blinded photographic assessment or detailed ethnic breakdown.^24^

Zhang et al described a single-center observational cohort of 50 female patients (mean age 58.7 ± 6.0 years; range, 46-75 years) who underwent a closed-neck facelift augmented by submental liposuction but without a submental incision. Through standard periauricular access, the surgeons performed a high SMASectomy, lateral platysmaplasty (platysmal window anchored to the mastoid fascia), a minimal-access cranial-suspension (MACS) malar lift, and central-face fat grafting. At a mean follow-up of 12 months (range, 14-81 months) patient-reported FACE-Q outcomes showed moderate satisfaction, with an average “Appraisal of Neck” score of 73.8 ± 9.0 and “Appraisal of the Area Under the Chin” of 71.0 ± 7.8 (0-100 scale; *n* = 50). The study did not detail perioperative complications or provide blinded photographic assessment, and its all-female, single-arm design limits generalizability; nevertheless, it suggests that a closed approach combined with selective liposuction and ancillary soft-tissue techniques can yield acceptable subjective neck and submental contour results at 1 year.^25^

Qiu et al presented an observational series of 20 East-Asian patients (mean age ≈51 years) who underwent a closed-neck facelift without liposuction. Through periauricular access, the surgeons executed a lateral SMASectomy, a MACS malar lift, and a set of purse-string SMAS sutures—collectively termed the “cable-stayed” technique. At a mean follow-up of 11.3 ± 9.8 months, FACE-Q assessment of the Lower Face and Jawline yielded a mean satisfaction score of 76.2 ± 14.9 (0-100 scale; *n* = 50). No hematomas, seromas, nerve injuries, or skin-edge complications were reported. Although these findings support the safety and aesthetic effectiveness of the cable-stayed facelift technique, the single-arm design, modest follow-up duration, and the absence of blinded photographic evaluation limit the strength of the conclusions.^26^

Mentz et al analyzed 40 consecutive patients who underwent a deep-plane facelift paired with an open-neck procedure that incorporated submental preplatysmal fat excision (liposuction), partial anterior digastric resection, and midline platysmal plication—thereby classifying the series as open with liposuction. Half of the patients (*n* = 20) additionally received the Bolster Equalization Suture Technique (BEST), in which 3 external quilting sutures were placed along the cervicomandibular groove, whereas the remaining 20 served as controls. Demographics were comparable between arms (BEST vs control: mean age 59 vs 57 years; women 85% vs 90%). At 6 months, FACE-Q scores (0-100 scale) demonstrated similar aesthetic benefit irrespective of quilting: mean Appraisal of Neck 76.5 ± 9.4 and mean Appraisal of Area Under the Chin 74.3 ± 10.2. No hematomas, seromas, skin-edge necrosis, or nerve injuries were observed; 2 cases (10%) of minor postauricular delayed healing in the BEST group resolved with local wound care. Thus, adding quilting sutures to an open, liposuction-assisted necklift was safe but conferred no measurable advantage in early patient-reported neck-contour satisfaction within this small, short-term series.^27^

La Padula et al conducted a prospective Level III pilot study of 36 transgender women (mean age 41 ± 5.3 years) who underwent a deep-plane facelift combined with an open, liposuction-assisted necklift. Through standard periauricular incisions and a submental access, the team removed subcutaneous and subplatysmal adipose tissue (and, when indicated, interdigastric fat), performed medial platysmaplasty with optional anterior-digastric plication, executed a lateral SMASectomy with SMAS-flap suspension, and placed a hemostatic neck net to mitigate hematoma risk. At 12 months, patient-reported FACE-Q scores were excellent, averaging 91.9 ± 4.2 for Appraisal of Neck and 87.1 ± 2.1 for Appraisal of the Area Under the Chin (0-100 scale; *n* = 36). Morbidity was minimal: one self-limited minor hematoma (2.8%); no infections or facial-nerve injuries were observed. Although these data suggest that a deep-plane, open-neck approach with targeted fat debulking yields very high early satisfaction and low complication rates in a young transgender cohort, the small sample size, lack of a control arm, and short-term follow-up limit the generalizability and durability of the findings.^28^

Kim et al reported a large single-center observational series of 1000 Korean patients (983 women, 17 men) who underwent a standard SMAS facelift through periauricular incisions; 240 of these cases included a lateral-only necklift with platysma closure but no submental incision or liposuction—classifying the neck component as closed without liposuction. The surgeons tailored SMAS management to the degree of jowl descent, performing SMAS dissection in 651 cases, SMAS plication in 215 and SMASectomy in 134; 143 operations were secondary or tertiary revisions. At 12 months, the 240 necklift patients recorded high patient-reported outcomes on the FACE-Q scale (0-100): mean Appraisal of Neck 86.2 ± 18.5 and mean Area Under the Chin 87.8 ± 15.1. Complications were described only as “very rare,” with isolated and unspecified instances of transient facial-nerve paresis, infection, hematoma, and flap necrosis; none required reoperation. Although the large cohort underscores the safety and satisfaction achievable with a closed, lateral-based technique, interpretation is constrained by the absence of a control group, the heterogeneous mix of SMAS maneuvers, and the lack of detailed, quantified complication data.^29^

For a detailed overview of postoperative complications reported in the included studies, please refer to Supplemental Table 1, which summarizes the types and rates of morbidity across the cohorts. Table 1 presents results of the Risk of Bias Assessment from each study included in this paper. Overall risk of bias was rated as high for 4 studies (44.4%) and low for 1 study (11.1%).

**Table 1.** Risk of Bias Assessment of Included Studies

Study	Patient selection	Exposure and outcome ascertainment	Completeness of follow-up	Reporting clarity	Overall
Kappos et al (2017)^21^	Moderate	Low	Low	High	Moderate
Sinno et al (2015)^22^	High	Moderate	Low	High	High
Han et al (2024)^23^	Moderate	Low	Low	Moderate	Moderate
Wang et al (2018)^24^	High	Low	Low	High	High
Zhang et al (2021)^25^	High	Moderate	Moderate	High	High
Qiu et al (2021)^26^	Moderate	Moderate	Moderate	Moderate	Moderate
Mentz et al (2023)^27^	Low	Low	Moderate	Low	Low
La Padula et al (2023)^28^	High	Low	Low	High	High
Kim et al (2024)^30^	Moderate	Low	Low	High	Moderate

Risk of bias was assessed using criteria adapted from Murad et al for observational studies across 4 domains: patient selection, exposure and outcome ascertainment, completeness of follow-up, and reporting clarity.^19^ Low = low risk of bias; moderate = unclear or partial risk of bias; high = high risk of bias.

Complications were generally uncommon and mild, typically resolving spontaneously or with minimal treatment. Serious events, such as permanent nerve injury or major wound problems, were rare. However, comparison across studies is limited by inconsistent complication reporting—some provided incomplete data, whereas others offered only vague or general descriptions. Standardized, transparent reporting of complications is needed to enable clearer and more reliable assessment of surgical outcomes.

## DISCUSSION

Facial and cervical rejuvenation remain central in aesthetic surgery because patients seek effective procedures with minimal downtime, scarring, and complications. To our knowledge, this meta-analysis provides the first comparative evaluation of patient-reported outcomes between open and closed platysmaplasty using the validated FACE-Q Aesthetic Module. Across 9 studies and 847 patients, it offers quantitative insight into the comparative effectiveness and patient-reported satisfaction associated with different cervicofacial rejuvenation techniques.

When submental liposuction was performed, no significant difference was found in chin aesthetic outcomes between open and closed approaches (closed: 78.3 vs open: 75.2; *P* = .129), indicating that effective submental fat reduction—regardless of access—is the primary determinant of chin satisfaction (Figure 2). These findings align with La Padula et al, Mentz et al, and Lin and Zhang, who achieved comparable contouring outcomes through both open and closed techniques.^22,24,26,27^ Thus, increased invasiveness through submental incisions does not necessarily yield superior chin contouring.

In contrast, neck aesthetic satisfaction in the liposuction subgroup favored open approaches. Patients undergoing open procedures reported significantly higher Neck-FACE-Q scores compared with closed procedures (mean 81.5 vs 77.5; *P* = .022; Figure 3). This advantage likely reflects the direct access open techniques provide for modifying deeper structures—including the platysma, digastric muscles, and submandibular glands—and for correcting vertical platysmal banding through maneuvers such as corset platysmaplasty, consistent with Ellenbogen and Karlin.^3,4^ Closed techniques rely on lateral platysma suspension, which applies a less direct vector of tension. Open surgery also permits visually confirmed adjunctive interventions, which may further improve results.

A noteworthy finding was that closed techniques without liposuction produced significantly higher FACE-Q satisfaction than open procedures with liposuction (chin: 86.4 vs 75.4; neck: 86.6 vs 81.5; Figures 4, 5). This may reflect selection bias—closed, no-liposuction approaches are typically offered to patients with minimal fat and good skin quality. Liposuction itself may also reduce satisfaction because of potential irregularities or fibrosis in patients with limited dermal elasticity. Expectation differences may play a role as well: patients choosing conservative procedures often anticipate modest results, whereas those undergoing open surgery with liposuction may expect dramatic changes and thus have higher satisfaction thresholds. Even small liposuction scars may further affect perception of outcome in the thin-skinned submental region.

Liposuction also functions as a confounder in many open procedures, making it difficult to isolate the effect of open access alone. Our findings suggest that removing submental fat is not, on its own, a reliable predictor of higher satisfaction, emphasizing the need to balance procedural invasiveness with expected benefits. Reporting of concomitant facial procedures was inconsistent across included studies. Where documented, additional interventions such as lipofilling/fat grafting, blepharoplasty, and dermabrasion/resurfacing were performed alongside facelift/neck rejuvenation in some cohorts, whereas many studies did not clearly report whether additional procedures were performed. This limits attribution of patient-reported FACE-Q scores solely to differences in neck approach. Furthermore, age and tissue quality strongly influence neck rejuvenation planning. In general, younger patients have better skin elasticity, so closed techniques (with or without liposuction) may achieve effective redraping, whereas older patients more often have laxity, platysmal banding, deeper fat, and/or gland prominence that favors an open approach with adjunct maneuvers. Because age was inconsistently reported and rarely stratified by technique in the included studies, we could not adjust for age-related confounding; therefore, differences in FACE-Q scores may reflect patient selection as much as technique effect.

Clinically, these results support patient-specific surgical planning. Younger patients with good skin elasticity, minimal submental fat, and mild-to-moderate laxity are well suited to closed techniques, with or without liposuction. Older patients or those with advanced cervical aging—platysmal banding, subplatysmal fat, or glandular hypertrophy—are more likely to benefit from open approaches despite their greater invasiveness. These conclusions align with narrative reviews recommending individualized, anatomy-based strategies and aging patterns.^29^

By incorporating FACE-Q, this meta-analysis supports the increasing emphasis on patient-reported outcomes in aesthetic surgery, consistent with Pusic et al.^8^ Although the chin and neck are contiguous, the FACE-Q domains capture slightly different patient-perceived concerns (submental fullness/definition vs broader cervical contour), and reporting varied across studies. Overall, the findings reinforce the value of an algorithmic, patient-centered approach to cervicofacial rejuvenation and provide quantitative evidence to guide surgical decision making. Prospective studies should stratify patients by anatomic severity and compare liposuction vs no liposuction within standardized facelift technique and surgeon setting. Future research should prioritize well-designed prospective studies, ideally randomized, with standardized protocols, consistent follow-up intervals, stratification by anatomical and demographic variables, and inclusion of scar-specific and objective aesthetic assessments.

### Clinical Implications

This systematic review and meta-analysis reinforces the need for personalized surgical planning based on patient anatomy, aging patterns, and aesthetic goals. Closed-neck techniques are beneficial to younger patients with mild-to-moderate laxity and minimal submental fat. In contrast, open-neck approaches remain essential for advanced cervical aging marked by significant platysmal banding, subplatysmal fat, glandular hypertrophy, or substantial skin laxity. Their direct anatomical access enables comprehensive correction, and although more invasive, these approaches are often necessary to achieve optimal cervicomental contouring.

Integrating validated patient-reported outcome instruments such as the FACE-Q enhances the quality and objectivity of aesthetic assessment, providing reliable data on patient satisfaction and guiding more tailored treatment decisions.

Overall, the findings support a flexible, patient-centered approach in cervicofacial rejuvenation, using evidence-based algorithms tailored to patient-specific characteristics to improve outcomes and satisfaction.

### Limitations

#### Statistical Limitations

The statistical analysis has several key limitations. The small number of eligible studies, influenced by publication bias and limited reporting (8 out of the 9 studies have a moderate-high risk of bias), reduces statistical power and generalizability. Significant inter-study and intra-study heterogeneity is likely. Inter-study variation is exacerbated by the frequent absence of dual cohorts with different surgical methodologies, preventing paired comparisons, which are likely reflecting surgeon preference. Several studies were excluded for not reporting both mean and SD, and most provided minimal surgical detail beyond broad technique descriptions. Intra-study heterogeneity also remains unaddressed because key confounders such as age, BMI, skin quality, and surgeon experience were inconsistently documented. Despite these constraints, this review offers a structured, quantitative synthesis of the best available evidence using validated patient-reported outcome measures.

#### Other Limitations

Several methodological and clinical limitations warrant consideration. First, postoperative FACE-Q follow-up intervals varied widely (3 months to >5 years), complicating comparisons because satisfaction changes with scar maturation, surgical refinement, and evolving expectations.

Second, there is an absence of preoperative FACE-Q scores in most included studies. Without baseline data, it is not possible to determine the true magnitude of improvement following surgery. Differences in postoperative FACE-Q scores between open- and closed-neck techniques may reflect preoperative baseline differences rather than true differences in surgical efficacy. Future studies should incorporate paired preoperative and postoperative FACE-Q assessments to allow meaningful comparison of treatment effect sizes.

Third, demographic reporting was inconsistent. Ethnicity was often undocumented, and most cohorts were predominantly female, limiting generalizability.

Fourth, although the FACE-Q Aesthetic Module is validated, it does not assess scar quality—a key factor for procedures involving submental incisions, where scar appearance can significantly influence satisfaction.

Fifth, studies showed marked heterogeneity in surgical technique and traction vectors, particularly the depth and extent of dissection (deep-plane, high/extended SMAS, SMASectomy, purse-string suspension, MACS/cranial suspension, and variable SMAS management with different traction vectors). These differences may influence cervical contour independently of neck access, limiting comparability between open and closed cohorts. Please refer to Supplemental Table 1 for detail on study surgical techniques. Across included studies, closed-neck rejuvenation (no submental incision) was generally performed through a periauricular approach with SMAS-based maneuvers (eg, SMAS plication/SMASectomy, deep-plane, MACS, or purse-string suspension) relying on lateral traction and, where described, mastoid fixation, with or without adjunct liposuction. In contrast, open-neck approaches (submental incision) were commonly combined with fat debulking and additional deep contouring maneuvers such as midline platysmaplasty/platysma plication, subplatysmal fat management, and/or digastric modification, with occasional gland-related procedures. Technique reporting (including traction vectors and adjunct maneuvers) was inconsistent across studies, contributing to heterogeneity and limiting direct comparability between groups. This heterogeneity may confound open-vs-closed comparisons and limits attribution of FACE-Q differences to neck access alone.

Sixth, all studies were observational and nonrandomized, increasing risks of selection bias, procedural variability, and confounding and limiting causal inference.

Seventh, patient-reported FACE-Q scores may reflect overall perceived facial rejuvenation rather than neck-specific change alone. In the included literature, concomitant procedures (eg, fat grafting/lipofilling, blepharoplasty, dermabrasion/resurfacing) were present in some cohorts and incompletely reported in others, preventing adjustment for co-interventions. Consequently, differences in FACE-Q outcomes between groups may be confounded by additional procedures performed at the time of surgery, and conclusions should be interpreted cautiously.

Additional issues include statistically nonsignificant but clinically suggestive trends, likely reflecting underpowered analyses; inconsistent reporting of age, despite its influence on technique selection; incomplete evaluation of liposuction, which may increase submental irregularities in nonobese patients; and lack of consideration of chin morphology or hyoid position, both crucial to surgical planning and cervicomental aesthetics. Most studies did not report anatomic severity or indications for selecting open vs closed approaches, limiting adjustment for selection bias.

Collectively, these limitations highlight the need for standardized protocols, consistent follow-up, scar-specific metrics, detailed demographic and anatomical reporting, and randomized or blinded study designs to strengthen evidence in neck rejuvenation surgery.

## CONCLUSIONS

This systematic review and meta-analysis identify notable differences in patient-reported outcomes between open- and closed-neck rejuvenation techniques. Closed approaches without submental liposuction produced the highest FACE-Q satisfaction scores, suggesting they are well suited to patients with mild-to-moderate aging and favorable anatomy. However, this likely reflects patient selection and heterogeneity rather than technique effect alone. In comparison, open techniques with direct fat excision and midline platysmaplasty remain essential for more advanced presentations.

These findings should be interpreted cautiously because of significant methodological limitations. The evidence is largely based on heterogeneous, noncomparative observational studies with variable techniques, reporting practices, patient selection, and concomitant procedures. Crucial anatomical factors—such as chin projection and hyoid position—were never reported, concurrent surgical procedures, inconsistent demographics, and the absence of scar-specific outcomes further limit applicability.

Overall, the results emphasize the need for individualized, anatomy-based surgical planning approach to neck rejuvenation supported by validated patient-reported measures.

## Supplementary Material

ojag140_Supplementary_Data
