# Supplementary material for: A Systematic Review and Meta-Analysis of the Role of Open Platysmaplasty in Facelift and Necklift Surgery: Comparative Outcomes of Closed- vs Open-Neck Rejuvenation With Liposuction Using FACE-Q Scales
Source: Aesthet Surg J Open Forum. 2026 Jul 2;8:ojag140. doi: 10.1093/asjof/ojag140 (PMC13398994; doi:10.1093/asjof/ojag140)
Supplement: ojag140_Supplementary_Data [file ojag140_supplementary_data.zip › Supplementary Table 1.docx]

**Supplementary Table 1.** Comparison of complication rates across studies.

| **Title** | **Year** | **Characteristics of Included Studies** | **Number of Patients** | **Age of Patients** | **Complications Type** |
| --- | --- | --- | --- | --- | --- |
| Kappos et al | 2017 | Facelift Technique: Face/Neck Lift (SMAS), peri-auricular  Neck Approach: closed neck  Liposuction: no liposuction  Adjunct Maneuvers/  Concomitant Procedures: Upper/lower blepharoplasty (28 patients)  Follow-Up Timepoint: chin 61 months (9-108 months) | 200 (178 returned the completed FACE-Q) | Mean: 63 years; Range: 54–85 years | Not reported |
| Sinno et al. | 2015 | Facelift Technique: Face/Neck Lift (high extended SMAS), peri-auricular  Neck Approach: Open neck  Liposuction: with liposuction  Adjunct Maneuvers/  Concomitant Procedures: NR  Follow-Up Timepoint: 61 months (3-72 months) | 105 approached; 53 completed (50.5% response rate) | Mean: 63.0 (SD: 6.1); Range: 50–80 | Not reported |
| Chung Hee Han et al. | 2024 | Facelift Technique: Face/Neck Lift (Extended Deep-Plane, peri-auricular)  Neck Approach: closed neck  Liposuction:   - Group 1 (32.3%): No laser liposuction - Group 2 (67.7%): Laser-assisted liposuction (1,064 nm Nd:YAG, SmartLipo) – lower face volume reduction   Adjunct Maneuvers/  Concomitant Procedures: NR  Follow-Up Timepoint: 12 months (no reported range) | 62 | Mean: 48.6 years; Range: 40-64 years | Submental Hardness: 11 (17.7%) — 5 (8.1%) resolved with triamcinolone or spontaneously  Early Asymmetry: 6 (9.7%) — all resolved spontaneously |
| Wang et al. | 2018 | Facelift Technique: Face/Neck Lift (Peri-auricular, SMAS plication, purse-string includes platysma, anchored to mastoid periosteum, malar fat pad elevation)  Neck Approach: closed neck  Liposuction: no liposuction  Adjunct Maneuvers/  Concomitant Procedures: NR  Follow-Up Timepoint: 12 months (6-24 months) | 138 | Mean: 55.2 years; Range 39-70 years | Total Complications: 8 (6.4%)  Hematomas: 3 (2.4%)  Skin Slough: 3 (2.4%)  Widened Scars: 2 (1.6%) |
| Zhang et al. | 2021 | Facelift Technique: Face/Neck Lift (peri-auricular, high SMASectomy, MACS malar lift, central fat grafting, lateral platysmal window sutured to mastoid fascia)  Neck Approach: closed neck  Liposuction: with  liposuction  Adjunct Maneuvers/  Concomitant Procedures: NR  Follow-Up Timepoint: 12 months (mean 14-81 months. No reported range) | 50 | Mean: 58.7 years ± 6.0; Range: 46-75 years | Not reported |
| Qiu et al. | 2021 | Facelift Technique: Face/Neck Lift (peri-auricular (SMAS plication with “cable-stayed” purse-string suspension and minimal access cranial suspension lift)  Neck Approach: closed neck  Liposuction: No liposuction  Adjunct Maneuvers/  Concomitant Procedures: NR  Follow-Up Timepoint: 11.3 +/- 9.8 months (no reported range) | 50 | Mean; 51 years | Hematoma,0 (0. %)  Seroma,0 (0.00 %) |
| Mentz et al. | 2023 | Facelift Technique: Face/Neck Lift (peri-auricular, partial anterior digastric resection)  Neck Approach: open neck  Liposuction: with liposuction  Adjunct Maneuvers/ Concomitant Procedures::   - BEST Group (n=20): With Bolster Equalization Suture Technique - Control Group (n=20): Without BEST   Follow-Up Timepoint: 6 months (no reported range) | 40 | BEST group: Mean 59 years (Range: 43-71)  Control group: Mean 57 years (Range: 42-70) | Postauricular Delayed Healing, 2 (10 %) - Resolved with local wound care |
| La Padula et al. | 2023 | Facelift Technique: Face/Neck Lift (peri-auricular, deep plane) Neck Approach: open neck  Liposuction: with liposuction  Adjunct Maneuvers/ Concomitant Procedures: hemostatic NET in neck  Follow-Up Timepoint: 12 months (no reported range) | 36 | Mean age: 41 ± 5.3 years.  No data on Median or Range is explicitly provided. | Hematoma,1 (2.78 %) - no surgical intervention |
| Kim et al. | 2024 | Facelift Technique: Face/Neck Lift (peri-auricular, SMAS plication 215 patients, SMASectomy 134 patients)  Neck Approach: closed neck  Liposuction: no liposuction  Adjunct Maneuvers/  Concomitant Procedures: NR  Follow-Up Timepoint: 12 months (no reported range) | 1000 (240 closed neck without liposuction) | 40 years and younger: 128; 50-60 years: 409; 60-70 years: 420; 70 years and older: 43* | Facial Nerve Injury, Infection, hematoma, flap necrosis- very Rare (number not reported) |

*Age distribution reflects the entire cohort of 1000 patients; data specific to the 240 applicable patients were not reported separately to the original publication
